# Exploiting Agri-Food Waste as Feed for *Tenebrio molitor* Larvae Rearing: A Review

**DOI:** 10.3390/foods13071027

**Published:** 2024-03-27

**Authors:** Konstantina Kotsou, Theodoros Chatzimitakos, Vassilis Athanasiadis, Eleni Bozinou, Stavros I. Lalas

**Affiliations:** Department of Food Science and Nutrition, University of Thessaly, Terma N. Temponera Str., 43100 Karditsa, Greece; tchatzimitakos@uth.gr (T.C.); vaathanasiadis@uth.gr (V.A.); empozinou@uth.gr (E.B.); slalas@uth.gr (S.I.L.)

**Keywords:** agri-food waste, *Tenebrio molitor*, proximate composition, nutritional value, larvae

## Abstract

The agri-food industry generates substantial amounts of waste, including by-products and residues. The increasing demand for sustainable and eco-friendly practices in the agri-food sector has sparked an interest in finding alternative uses for such waste materials. One promising approach is the utilization of waste from the agri-food industry as feed for the rearing of mealworms (*Tenebrio molitor*). Since agri-food waste is rich in proteins, carbohydrates, lipids, and vitamins, as well as other bioactive compounds, all of which are essential for insect growth and development, incorporating such waste into the diet of mealworms promotes sustainable insect production, reducing the economic and environmental problems associated with waste disposal. This practice can also be beneficial for the rearing of mealworms since their nutritional value can also be enhanced. To this end, various waste materials, such as fruit and vegetable peels, spent grains, and food processing residues, have been investigated as potential feed sources, leading to increased mass production, lower cost, and enhanced nutritional value. This review aims to highlight the potential of agri-food waste as a feed source for mealworms, as well as their potential to enhance their nutritional value. Furthermore, the potential applications of mealworms reared on agri-food waste are highlighted, including their potential as a sustainable protein source for human consumption and as feed ingredients in the livestock and aquaculture sectors.

## 1. Introduction

The practice of eating insects may not be a new habit since humans have been consuming them for thousands of years [1,2,3]. Europe, traditionally less inclined towards insect consumption, has witnessed a significant shift in the past years with the continuous approval of insects, like *Locusta migratoria* (Orthoptera: Acrididae)*, Tenebrio molitor* (Coleoptera: Tenebrionidae), *Alphitobius diaperinus* (Coleoptera: Tenebrionidae), and *Acheta domesticus* (Orthoptera: Gryllidae), for human consumption [4,5,6,7,8]. Moreover, for over five years now, Europe has been granting approval and promoting insect farming and utilization, particularly in the realm of animal feed. In more detail, eight insects, namely *Hermetia illucens* (Diptera: Stratiomyidae), *Musca domestica* (Diptera: Muscidae), *Tenebrio molitor* (Coleoptera: Tenebrionidae), *Alphitobius diaperinus* (Coleoptera: Tenebrionidae), *Acheta domesticus* (Orthoptera: Gryllidae), *Gryllodes sigillatus* (Orthoptera: Gryllidae), *Gryllus assimilis* (Orthoptera: Gryllidae), and *Bombyx mori* (Lepidoptera: Bombycidae), have been approved for use as animal feed for aquaculture, poultry, and swine [9,10]. *Tenebrio molitor* (TM) is the most widely accepted insect for rearing and consumption in Europe [11,12,13,14]. This explosion of scientific interest can be traced to two strong considerations.

The noteworthy nutritional profile of ΤΜ is the first and foremost reason for its high acceptability. It is especially highlighted for having a remarkable quantity of crude protein, essential amino acids (EAAs), crude fat, essential fatty acids (EFAs), and a variety of minerals [15,16,17,18]. In particular, its protein content ranges from 13.7 to 60.2% [19,20], whereas its fat content fluctuates from 17.0 to 40.0% [21,22]. Furthermore, it boasts all of the EAAs (histidine (His), isoleucine (Iso), leucine (Leu), lysine (Lys), methionine (Met), phenylalanine (Phe), threonine (Thr), tryptophan (Trp), and valine (Val)) [23,24,25,26,27,28] and EFAs (myristic acid (C14:0), palmitic acid (C16:0), palmitoleic acid (C16:1), and omega-9 oleic acid (C18:1)) [29,30,31,32,33,34,35]. Last but not least, the following minerals have been identified and quantified in TM: 1.0% phosphorus (P) [36], 0.5% calcium (Ca) [20], 0.4% sodium (Na) [37], 1.6% magnesium (Mg) [20], 1.0% potassium (K) [37], 100.02 mg/kg of iron (Fe), 117.4 mg/kg of zinc (Zn), and 20.0 mg/kg of copper (Cu) [36].

The second reason for the high preference for TM lies in the several ecological benefits that the rearing of TM larvae offers. TM growth and development require limited land use and unexpansive facilities, especially when compared to conventional livestock farming [38,39,40], making them a cost-effective option. Moreover, TM larvae are well-known for their low water footprint owing to the fact that they require minimum amounts of water for their growth and development, unlike many other livestock animals [41]. Greenhouse gas emissions are one of the main reasons climate change exists, and traditional livestock like cattle or poultry contribute negatively to this [42,43]. On the other hand, TM larvae, due to their cold-blooded characteristics [44,45], do not engage in activities that lead to significant methane emissions, which contribute significantly to the large environmental footprint of agriculture [46]. Finally, TM larvae play a crucial role in a circular economy model because they can grow and develop properly with organic waste streams, such as agricultural by-products, as their feed [47,48,49], leading to a solution to the major problem of sustainable exploitation of the large amount of agricultural waste produced each year.

It is estimated that more than 1.3 billion tons of food waste (13.8% of global food production) are generated annually worldwide [50], with European Union countries responsible for 90 million tons of agricultural waste annually [51]. This astounding amount of waste has significant environmental, economic, and social consequences [52,53], since the global food system is a major contributor to land use change, environmental degradation, and resource depletion and causes significant greenhouse gas emissions through food production, processing, and disposal [54]. For this reason, this issue was added to the 17 Sustainable Development Goals (SDGs) concerning “sustainable consumption and production patterns”. More specifically, according to goal 12.3, global per capita food waste should be halved by 2030 (within 6 years from now) [50,55]. Therefore, improvements in consumer education and the adoption of waste management strategies are vital. The ecological benefits of raising TM make them an attractive option for sustainable protein production, particularly in a world facing challenges related to agri-food waste exploitation. 

Given the comprehensive information provided and a thorough examination of the existing literature, this study seeks to examine the potential benefits, such as a possible increase in nutritional content, as well as the drawbacks, such as potential viability issues, associated with rearing TM larvae using agricultural and food waste. Furthermore, the possibility of substituting or supplementing traditional feed for TM larvae with agricultural waste in order to facilitate a swift and cost-effective increase in the nutritional value of the larvae along with a reduction in food waste is examined. As such, emphasis is placed on the effect of various diets on the growth and nutritional value of the larvae in light of establishing a robust circular economy system where waste materials are repurposed. 

## 2. Theoretical Background

As stated above, rearing TM larvae is both convenient and economical. TM larvae are reared in controlled-conditions cabinets [56], usually in plastic or glass vials or containers with a cover. Sufficient ventilation holes in the lid are very important to ensure proper air circulation [57,58]. Moreover, for adequate growth and development of the larvae, specific conditions of temperature and relative humidity and, at the same time, constant darkness are required [59,60]. In more detail, TM larvae are recommended to be reared at high temperatures, in a temperature range of 25–27.5 °C [15,61,62]. Furthermore, a very high relative humidity is also required for proper rearing, with an optimum range between 60 and 75% [56,63,64]. However, despite the high relative humidity required in the environment for the growth and development of larvae, low moisture is required within the feeding substrate [65]. Previous studies have shown that by adding different moisture sources within the feed substrate, for example, fresh carrot, fresh potato, and agar, the larvae showed better growth, had a higher weight, and completed their development faster than those without additional moisture sources [37,66,67,68]. However, for reasons of general hygiene, maintaining a source of moisture within the feed substrate should be carefully managed to prevent signs of mold [69]. In addition, the growth, development, and subsequent nutritional value of the feed substrate are of great importance. TM larvae are recommended to be reared on starchy substrates, mainly flour and wheat bran [28,70,71,72]. However, many studies have been carried out recently that examined the inclusion of other products as constituents in the rearing of TM larvae. Most of the ‘new’ substrates are prepared in various proportions and mixed with their conventional feed. The addition of the extra constituents is carried out in order to enhance the high nutritional value of the insects and reduce the overall cost. Such additives in the insect diet include oyster shell flour, which contains 8% calcium (Ca), used to boost TM larvae with Ca [73], and Moringa oleifera leaves, abundant in protein [74], polyphenols [75], flavonoids [76], β-carotene [77], and vitamin C [75], used for the overall nutritional enhancement of TM larvae. Lastly, avoiding overcrowding and maintaining the right density of larvae is essential for preventing competition for feed and space [78,79].

## 3. Materials and Methods

Regarding the methodology used in this review, three electronic databases, namely Google Scholar, Scopus, and ScienceDirect, were employed to conduct a comprehensive search for research studies. The exploration of alternative by-products for the rearing of TM larvae represents a novel research field initiated approximately 12 years ago. The chronological scope of the cited investigations ranges from 2012 to 2024 in order to provide a comprehensive overview of all tested rearing conditions, comparing or contrasting older studies with more recent ones. In this review article, all available articles are included, whether provided free of charge for reading or not. The search terms used to identify articles published within the aforementioned time frame were as follows: (“Tenebrio molitor” OR “larvae” AND “rearing” OR “growth” OR “development” OR “increase” OR “nutritional value” AND “waste” OR “by-products”). The results in all articles were carefully examined for relevance to the review topic, and only suitable ones were presented.

## 4. Results and Discussion

**Figure 1:**
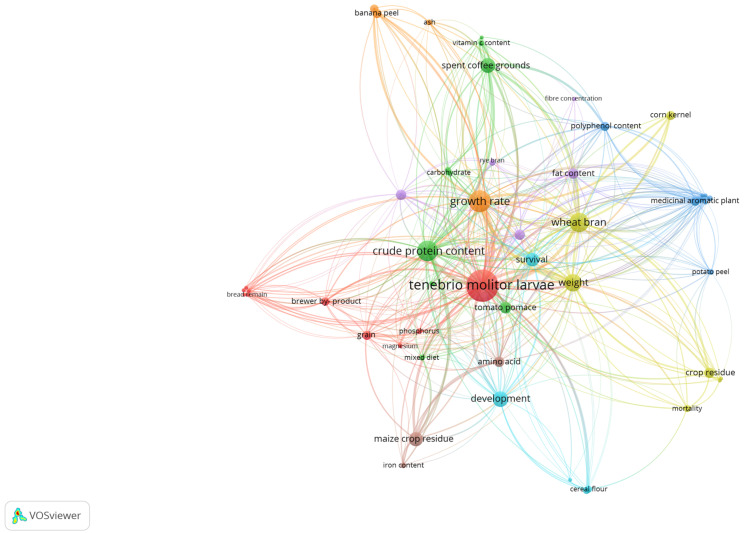
Visual representation (developed by VOSviewer version 1.6.20) of the relationships between different parameters related to agri-food waste used as feed for TM rearing.

**Figure 2:**
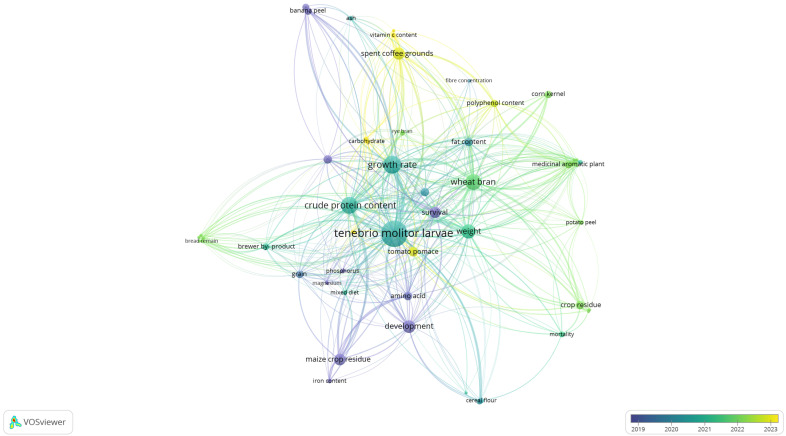
Chronological presentation of the studies on the topic of agri-food waste employment as feed for TM rearing.

The evaluation of new economical, environmentally friendly, and nutritious substrates for the diet of TM larvae is a hot topic in the scientific community and a highly promiscuous field for industrial applications. Many research articles have been published thus far, and a plethora of by-products and other food wastes have been tested and assessed thus far. These products range from fruits, vegetables, and grains to edible fungi and beverages, and combinations of the above have also been explored. Figure 1 and Figure 2 provide a visual representation of the relationships discerned from the data analysis by VOSviewer that showcase the relationship between diverse substrates used for the rearing of TM larvae and the nutrient factors that were studied, impacting their growth and development. 

In Figure 1, it can be seen that wheat bran was extensively used, and the larvae were studied in terms of nutritional value and growth. This was somewhat expected, given the fact that wheat bran is an established diet for TM larvae, alongside other starch-rich products. Also, it can be seen that some crop-related by-products are less related to many nutritional value parameters, unlike maize crop residue and spent coffee grounds, which demonstrate more relationships with nutritional value parameters. Figure 2 depicts a chronological presentation of the abovementioned relationships. It can be seen that in this field of research, initially, many crop-related by-products were studied in terms of larval survivability and growth without much emphasis on nutritional value. More recent research focuses on other food by-products, such as spent coffee grounds and tomato pomace, while more nutritional parameters are being examined, such as vitamins (e.g., vitamins C and A), carotenoids (e.g., lycopene), and polyphenolic compounds. This depiction highlights a multifaceted approach towards maximizing the nutritional value of TM larvae, encompassing both conventional dietary sources and innovative utilization of agricultural by-products to enrich their nutritional profile comprehensively. As such, a comprehensive overview of current studies is needed in order to guide further research endeavors. In this article, for ease of presentation and reading, each category of by-product will be discussed separately. Also, since in some cases, multiple efforts have been made to examine similar by-products, the studies regarding the same by-product are presented in chronological order.

### 4.1. Fruit By-Products as Feed Substrate for Tenebrio molitor (TM) Larvae

A tomato is a fruit that belongs to the category of berries [80] and is an integral part of the Mediterranean diet [81]. Roughly 4% of the tomatoes end up as waste after post-harvest processing [82]. Zim et al. [83] decided to exploit the cultivation residues of tomatoes and include them in the diet of TM larvae in order to test their effect on growth and development. A combination of wheat bran (60%) and starter feed for broiler chickens (40%) was applied as a control diet, while tomato residue was used either as a complete feed or in combination with wheat bran (tomato residue 50% + wheat bran 50%). Combining tomato residue with bran was referred to as toxic because it did not increase the growth of the larvae, and the mortality rate was extremely high, reaching almost 100%, without further exploiting the reasons for this high mortality rate. However, using diets with 100% tomato residues presented promising results in terms of viability. In particular, the viability was similar to that of the control sample and approached 90%. Notwithstanding, the weight of larvae reared on a 100% tomato residue diet showed low levels, recording 1 g weight, while the larvae in the control sample recorded 3 g, 200% higher weight. Similar results were recorded in the growth rate, where larvae consuming 100% tomato residue in their diet recorded a growth rate of just over 1%, while the control sample exhibited a growth rate of about 7.5%. Thus, it could be inferred that tomato residues were not a viable option for TM rearing. Later on, the study conducted by Baldacchino et al. [84] provided a better overview of the impact of tomato pomace on TM rearing. Specifically, four dietary substrates were assessed, comprising: (1) 100% wheat bran, (2) 73% wheat bran with 27% tomato pomace, (3) 59% wheat bran with 41% tomato pomace, and (4) 100% tomato pomace. One of the most intriguing findings was that the larvae exhibited a pronounced preference for consuming tomato pomace, with an increase in its inclusion leading to a corresponding rise in feed consumption, culminating in a feed consumption (FC) of 245.7 mg per individual larva. While diets containing tomato pomace appeared palatable to the larvae, they did not favorably influence their protein profiles. Larvae reared on diet 1 exhibited approximately 50% crude protein content, whereas those reared on diet 4 demonstrated approximately 40% crude protein content, signifying a reduction of protein content up to 20%. Additionally, it is noteworthy that tomato pomace is rich in antioxidant bioactive compounds, such as lycopene and β-carotene [85]. The results indicated that an increase in the content of tomato pomace in the dietary substrate led to a proportional augmentation in these bioactive constituents within the TM larvae. Specifically, in TM larvae, the quantity of lycopene escalated from 0.10 µg/g (diet 1) to 1.20 µg/g (diet 4), manifesting a 91.67% increase. Simultaneously, the content of carotenoids in the larvae increased by 80.82% (diet 4). Therefore, despite tomato by-products not being considered the most suitable dietary substrates for TM rearing, this research demonstrated opposing results by reporting not only a preference of TM for tomato by-product larvae but also an enhancement of several nutritive parameters. Lycopene, as an antioxidant, inhibits the autoxidation of lipids and related products [86], while beta-carotene serves as an antioxidant and anti-inflammatory agent, bolstering immunity and augmenting intracellular signaling [87]. As such, increasing their content in the larvae will result in health benefits for consumers. 

Zim et al. [83] also studied citrus residues besides tomato waste. The pulps, peels, seeds, and membrane citrus residues, which make up 40–60% of citrus fruits, collectively surpass approximately 110–120 million tons of waste annually [88]. The experimental procedure followed the same pattern as for tomato residues, that is, two diets were tested, one containing 100% citrus residue and one containing 50% citrus residue mixed with 50% wheat bran, and both diets were compared with a control diet containing wheat bran 60% and starter feed for broiler chicken 40%. The diet of the combination of bran with citrus residue caused approximately 30% mortality, while the feed comprising citrus residue solely caused approximately 80% mortality (considered toxic). However, no further insights were provided to justify the rise in the mortality rate. In addition, despite the low survivability of the sample reared with 50% citrus residues mixed with 50% bran, larval weight and growth rate did not increase significantly. As far as the weight of the larvae is concerned, the sample reared with 100% citrus residue weighed more than 2 g, while the control sample weighed about 3 g. Outstanding was the growth rate of TM larvae reared with 100% citrus residue, wherein it exhibited a growth rate of more than 5%, with the control sample recording 7.5%. Hence, this makes it evident that citrus crop residues are a promising by-product that could be further studied for rearing TM larvae. The latest research on the use of fruit sub-products for enhancing the nutritional value of TM larvae was carried out by Kotsou et al. [89]. Specifically, various percentages of orange albedo (the inner part of the peel) mixed with wheat bran were used as nutritional substrates. Three diets with dried albedo used 10% albedo mixed with 90% wheat bran, 17.5% albedo mixed with 82.5% wheat bran, and 25% albedo mixed with 75% wheat bran. Larvae reared on a 100% wheat bran diet were used as a control sample. The study indicated that as the percentage of albedo in the diet increased, the nutritional profile of the larvae was enhanced. Notably, the crude protein percentage exhibited up to a 32% increase since the control sample had a protein content of 32.25%, while the sample reared with 25% albedo had a protein content of 44.20%. In addition, the percentage of ash also increased from 1.06% (control sample) to 1.34% (TM larvae reared with 25% albedo as a food substrate), thus resulting in an increase of 26.5%. The major nutritional reinforcement was in vitamins A and C, which, as mentioned in the introduction, occurred in small amounts in TM larvae. Vitamin A increased by 198% while vitamin C increased by 46% between the samples previously compared. Such an increase may be due to the fact that orange albedo is rich in these two vitamins [90], demonstrating the hypothesis that nutrient-rich dietary substrates lead to nutrient-enhanced TM larvae. Finally, a corresponding result of utmost importance is that the use of albedo as a nutritional substrate augmented the polyphenol content and antioxidant activity of TM larvae. Polyphenol content increased by 31.8% between the control sample and the sample of the diet containing 25% albedo, where values of 59.67 to 78.67 mg/g were recorded. In agreement with the previous results were the results of antioxidant activity, where the antioxidant activity increased from 119.39 μmol AAE/g (control sample) to 145.97 μmol AAE/g (larvae reared with 25% orange albedo).

Another attempt to enhance the growth and nutritional value of TM larvae was carried out by Loh et al. [91], who tested diets that contained watermelon rind. Edible rind makes up about 40% of the total mass of watermelon, resulting in approximately 36 million tons of waste watermelon rind annually [92,93] (about 67% less waste compared to citrus residues). Watermelon rind has been recorded to have 11.17% protein content and 13.09% ash content [94], while being found to be rich in vitamins such as C, A, and B complex vitamins [95]. In order to provide a standard for the validity of the experiments, TM larvae were reared with bread and used as a control. The crude protein content of the control larvae was 41.51%, while in the TM larvae reared with watermelon rind, the crude protein content was 43.38%, that is, the latter having an enhanced protein content of 4.50%. The use of watermelon rind also enhanced the ash content since the content of ash increased from 1.87% to 4.40%, that is, a 135.29% increase. This signifies that the use of a nutrient-rich component as feed for TM larvae can increase the content of the insect in the respective nutrient. Interestingly, apart from enhancing the nutritional value, the consumption of watermelon rind by TM larvae also enhances the length and weight gained by TM larvae. Specifically, in the control sample, a length gain of 18.30% was recorded, while in TM larvae consuming watermelon rind, the corresponding length was 22.17%. Moreover, in terms of weight, in the control sample, the gained weight of the larvae was 90.67%, while in the tested sample (watermelon rind as a feed substrate), the corresponding weight was 102.26%. Therefore, the application of watermelon rind can constitute an ideal feeding substrate for TM larvae, providing them with enhanced nutrients and improved growth and development.

Bananas are among the most marketable fruits in the world [96]. In fact, they are the second most produced fruit after citrus fruits, accounting for 16% of global fruit production, and the fourth most important food crop after rice, wheat, and maize [97]. India (30.4 million tons of production), China (11.6 million tons), Indonesia (7.2 million tons of production), and Brazil (6.8 million tons of production) are the four main countries where the cultivation of banana trees takes place [97,98]. This massive production results in generating large amounts of waste (36 million tons of banana peel) [99] since the peel part is not usually used except as organic fertilizer or animal feed [100]. At least two years are required for the decomposition of banana peels, leading to the production of excessive emissions of greenhouse gases [97]. Dried banana peel has been found to contain low protein levels (6.00–9.00%), suggesting low nutritional value [99]. This was also the case when banana peel was used for rearing TM larvae, since no increase in the protein content of the larvae was observed. Specifically, two TM larval-rearing diets were tested by Loh [91], one consisting of bread (the control sample) and the other of banana peels. In the control sample, the protein content was 41.51%, while in the samples of the banana peel diet, the protein content of the larvae was 38.53%, an amount reduced by 7.73%. Furthermore, the consumption of banana peels by TM larvae did not enhance their development either. In other words, the length gained during their growth in the banana peel was 13.90%, while the corresponding length in the control larvae was 18.30%. Similarly, the weight increase of the larvae was also lower in the samples with bananas in their diet. A weight increase of 64.76% was recorded in the banana diet-fed TM larvae and 90.67% in the control diet-fed larvae. However, the mineral content of the larvae seemed to increase upon consumption of banana peels in the diet, as it was elevated by 32.62%. This increase may possibly be due to the increased mineral content of the peels, such as phosphorus, iron, calcium, magnesium, and sodium [99]. Therefore, banana peels may not favor the growth and nutritional development of TM larvae, but they can enhance their mineral content.

### 4.2. Vegetable By-Products as Feed Substrate for Tenebrio molitor (TM) Larvae

Potatoes are a very popular food worldwide, preferred by all ages, and consumed in many different ways [101,102]. The main five potato-producing countries are Germany, France, the Netherlands, the United Kingdom, and Belgium, which collectively account for 60% of the total world cultivation [103]. Similar to other vegetables, after the potato is harvested, various residues are produced that remain unexploited [104]. Zim et al. [83] used this residue as a substrate for feeding TM larvae, with the aim of creating a circular economy system. Two percentages of potato residues were tested: a 100% potato residue and a 50% potato residue, while a combination of wheat bran 60% and starter feed for broiler chickens 40% was used as a control sample. As in the case of tomatoes, the combination of potato residues and bran did not seem to promote the viability of the TM larvae since the mortality rate was again high, reaching up to 90%. In the case of 100% potato residue, more promising results were recorded. In particular, the weight gained by the larvae was close to 2 g, while the larvae of the control sample were at 3 g. In terms of growth rate, TM larvae fed on potato residues had a growth rate of about 4%, while the control sample larvae had a growth rate of 7.5%. It is perceived that despite the potato residues had a positive impact on the growth and development of the larvae, they did not record values close to those of the control sample. Nevertheless, reevaluation of the above residues or examination of other percentages is suggested. 

Global potato production is 359 million tons per year, while approximately 16% to 25% of the potato weight is wasted during processing, resulting in ~89 million tons of waste [105]. A worrying fact is that the amount of waste is estimated to increase by 8 million tons by 2030, with associated greenhouse gas emissions of 5 million tons of CO_2_ equivalent, resulting in an eminent problem that needs to be addressed [102]. As such, exploitation of potato by-products attracts the interest of several researchers. To this end, Andreadis et al. [47] proposed the exploitation of potato peels for TM rearing. Along with the control and test feed (containing potato peels), two percentages (10 and 20%) of essential oils from medicinal aromatic plants were added to each substrate, resulting in a total of six feed substrate samples: one with plain wheat bran—the control sample (WB0), one with 10% essential oil added to the wheat bran (WB10), one with 20% essential oil added to the wheat bran (WB20), one with potato peels (PP0), one with potato peels and 10% essential oil (PP10), and one with potato peels and 20% essential oil (PP20). In the samples where essential oil was added, viability seems to be highly supported, with the addition of 10% being the most appropriate addition. Specifically, WB0 had 37% TM larvae viability at the end of growth, WB10 had 82% TM larvae viability at the end of growth, and WB25 had 72.6% TM larvae viability at the end of growth. Meanwhile, regarding the potato peel samples, at the end of growth, PP0 showed 25% TM larvae viability, PP10 showed 44.8% TM larvae viability, and PP25 showed 44.8% TM larvae viability. Referring to the protein profile of the larvae in the control samples, the addition of oil from medicinal plants does not significantly affect the protein content of the larvae. Specifically, the crude protein content of sample WB0 was 51.20%, WB10 was 52.10%, and WB25 was 50.60%. However, TM larvae reared on the diet of potato peels without the addition of oil also had the highest protein content, reaching 59.50%, that is, an increased protein content of 14.2% compared to the highest value recorded in the control samples. Remarkably, the addition of essential oil percentages significantly reduced this amount, as in the PP10 sample, the percent of the crude protein content of the samples was 44.10%, while in the PP25 sample, the crude protein content of the samples was 43.60%, a reduction of 34.92 and 36.47%, respectively. In terms of polyphenol content, sample WB0 showed the highest value, about 1.70 mg GAE/g DW, while in terms of potato peel samples, a higher polyphenol content was found in the sample where 20% essential oil was added, and its value was close to 1.30 mg GAE/g DW. As far as the flavonoid content of the samples is concerned, samples PP0, PP10, and PP25 also showed the highest values, which greatly exceeded the larvae content in the control samples. In particular, regardless of the addition of essential oil, the flavonoid content was close to 1.00 mg GAE/g DW, while the highest amount was recorded in PP10, which was close to 2.50 mg GAE/g DW. The fact that the substrates consisting of potato peel had the richest flavonoid-rich TM larvae may be due to the fact that potato peel is rich in phenolic compounds [106].

In addition to potato residues, TM larvae were also tested for the same reason in pepper culture residue by Zim et al. [83]. Two diets with pepper were used: 100% pepper residue and 50% pepper residue with 50% wheat bran. These diets were tested for their suitability by comparing viability, weight, and growth rate against a control sample consisting of wheat bran 60% and starter feed for broiler chicken 40%. Pepper residue in the larval diet at 50% appeared to be an unsuitable feed for TM larvae since larval mortality reached 50% with weight and growth rate reaching sub-zero levels. As far as the use of 100% pepper residues in larval feeding is concerned, it is evident that the results are the same as the experimental procedure using potato residues. Specifically, the weight gained by the larvae was close to 2 g, while the larvae of the control sample were at 3 g, that is, 50% heavier than the TM larvae reared on the control diet. The growth rate of TM larvae reared on a pepper residue diet was about 4%, while larvae reared on wheat bran 60% and starter feed for broiler chicken 40% (control sample) had a growth rate of 7.5%.

### 4.3. Grain/Cereal By-Products as Feed Substrate for Tenebrio molitor (TM) Larvae

Spent grains, apart from being available all year, constitute an economic by-product. Also, spent grains are a nutritious by-product as they are rich in fiber, protein, and minerals [107,108,109]. Presumably for the above reasons, Mancini et al. [110] decided to implement these sub-products for the rearing of TM larvae and to test the effect on their nutritional value. The larvae were reared with spent grains in two proportions, 100% (sample 1) and 50% spent grains mixed with 50% biscuits (sample 2), and showed the following results. The crude protein percentage for sample 1 was 17.36%, and for sample 2, it was 17.65%, that is, they exhibited a similar protein profile. In addition, the percentage of ash in samples 1 and 2 was 1.10 and 1.07%, respectively, showing a difference of only 2.8%. Still, the estimated carbohydrate content of samples 1 and 2 was 8.40 and 7.05%, respectively. At this point, it is worth emphasizing the value of carbohydrates for the human body for a balanced diet to be followed. Furthermore, the antioxidant activity of the TM larvae of the two diets was also evaluated in three different ways, ABTS reducing activity; 1,1-diphenyl-2-pircydrazyl, DPPH radical scavenging activity; ferric reducing ability (FRAP). According to the results, in the first method (ABTS), sample 1 recorded a value of 1.73 mmol Trolox equivalent/kg, and sample 2 recorded a value of 2.01 mmol Trolox equivalent/kg. Moreover, with the DPPH method, samples 1 and 2 recorded values of 0.35 and 0.30 mmol Trolox equivalent/kg, respectively, while using the FRAP method, they scored values of 0.75 and 0.86 mmol Trolox equivalent/kg, respectively. Meanwhile, the samples were also evaluated for their tocopherol content, where the total tocopherol content in sample 1 was 0.79 mg/kg and 3.16 mg/kg in sample 2, demonstrating that sample 2 was richer in antioxidant substances [111].

The crude protein and iron content of TM larvae reared on a diet containing wheat bran, oats, brewer’s yeast (50:45:5% by weight), a control sample, and TM larvae reared on a diet containing organic maize stover dry (chopped) (100%) were studied by Stull et al. [24]. The samples completed their growth in the same period of time, 32 days, but with very different weights. In particular, although the samples initially had similar weights (12.20 and 12.72 mg/g, the control sample and the sample reared with corn by-products, respectively) at the end of growth, in the control sample, every larva had a weight of about 128.94 mg/g, whereas each larva reared on maize by-products had a weight of 64.01 mg/g larva, that is, in the control sample, the larvae were 101.44% heavier. In addition to the reduced weight gain, larvae reared on organic maize stover dry showed a much lower crude protein content. Specifically, the larvae had a weight of 15.17%, while larvae reared with the control sample had 19.33% protein content, a 27.4% reduction in protein content. This reduction in protein content may be due to the low weight of the larvae at the end of their development. Although it is evident that corn by-products did not favor TM larval rearing content, the iron content of the larvae in this diet was higher than the control sample. The control sample contained 0.0157 mg iron/g larva, while the sample to be evaluated contained 0.0169 mg iron/g larva, thus showing an increase of 7.64%, a promising rate for the production of iron-rich larvae. Iron is one of the most important metals, and besides being required for human development, the human body uses iron to produce hemoglobin and certain hormones [112,113].

Brewer’s spent grain is the major by-product of the cereal industry, accounting for about 85% of the total by-products produced [114]. As mentioned previously, the brewer’s substrate was used at a relatively low percentage (5%) in the diet substrate of TM larvae used as a control sample [24]. However, years ago (2012), a different brewer’s product was tested for its effect on the growth and development of TM larvae and their nutritional value [115]. The following were tested: 100% wheat bran—control sample, 100% brewer’s spent grain—sample 2, 100% distillers dried grain—sample 3, 50% brewer’s spent grain and 50% wheat bran—sample 4, and 50% distillers dried grain and 50% wheat bran—sample 5. Regarding the completion of growth (transfer to pupae), the larvae from all samples completed their growth in approximately the same time of 6.5–7 weeks. Regarding the crude protein content of the larvae, samples 1 and 3 yielded exactly the same amount of 29.31%, followed by sample 5 with 22.30%. Nevertheless, the larvae reared with sample 2 had the highest crude fiber content (17.70%), followed by sample 5 with 10.52%, while the control sample had 6.36%, 178.30, and 65.41% less than samples 2 and 5, respectively. Crude fiber is an advantageous nutrient due to the fact that it prevents or even cures constipation, diverticulosis, and coronary artery disease [116]. An additional positive result is that no dangerous microbes such as *Escherichia coli* O157:H7 and *Salmonella* spp. were found in any of the larval samples, nor heavy metals except mercury, which was found in samples 1 and 5 in amounts of 0.01 and 0.04 mg/kg. It is therefore concluded that all of the above sub-products could be used for rearing TM larvae, each offering enhancement in different nutrients, depending on the requirements of the individual feeder.

A few years later, Melis et al. [117] re-evaluated dried brewers’ spent grains as a feeding substrate for TM larvae and compared it with a control sample of larvae reared on 100% wheat bran. These samples were evaluated for protein, fatty acids, and ash content. The test sample was superior to the control sample in terms of crude protein, fat, and important fatty acids such as palmitic acid, omega-3, and omega-6. Specifically, larvae reared on dried brewers’ spent grains recorded 22.45% crude protein and 7.3% fat content. They were also 22.97% richer in palmitic acid than the control sample (17.85% content). In addition, larvae reared on dried brewers’ spent grains were 2.56% (55.19% content) higher in omega-6 fatty acids and 40.45% (6.91% content) higher in omega-3 fatty acids. Omega-3 fatty acids are among the most crucial fatty acids since they help with brain functions such as increasing the ability to learn and memorize and improving blood flow to the brain [118]. Therefore, dried brewers’ spent grains may eventually be a highly beneficial dietary substrate for TM larvae. Moreover, due to the fact that the substrate consisted of 100% dried brewers’ spent grains, the circular economy is also promoted to a great extent since the waste from the grain production sector is a favorable feed for the food sector.

Brewers’ substrate constitutes a ubiquitous nutritional substrate for the rearing of TM larvae since it was studied again in 2022 by Montalbán et al. [70]. The feed substrate consisted of bread remains and brewer’s yeast, which was highly concentrated in starch (61.1%) and protein (18.5%). TM larvae rapidly completed their development (formation of the first pulp by the reared larvae) within about 89 days. The weight of each larva was close to 60 mg, and the larvae also showed high viability (84.1%). Moreover, the larvae showed high protein content, reaching 45.96%, a percentage considerably high compared to the results recorded by previous studies (15.17%) [21], but in which a meager percentage (5%) of brewer’s yeast was used. Regarding the nutritional profile of TM larvae in macrominerals, the following values were recorded: 0.06% Ca, 0.72% P, 0.29 Na, 0.16% Mg, and 1.00% K. Finally, regarding the microbiological profile of TM larvae, seven phyla were identified in the microbiota of TM larvae. In particular, 67.88% Tenericutes, 6.04% Proteobacteria, 23.62% Firmicutes, and 1.86% Cyanobacteria were quantified, along with small amounts of Fusobacteria (0.04%), Bacteroidetes (0.33%), and Actinobacteria (0.23%).

Andreadis et al. [47] studied the effect of two cereals as dietary substrates on the growth and nutritional value of TM larvae. The cereals tested were rice bran and corn cob. These cereals were tested either in their original form or with the addition of various percentages of essential oil at 10 and 20%. This resulted in six different products: TM larvae raised with rice bran without the addition of essential oil (RB0), raised with rice bran and a 10% addition of essential oil (RB10), and raised with rice bran and a 20% addition of essential oil (RB20). Additionally, the following samples were also tested and analyzed: larvae raised with corn cob without the addition of essential oil (CC0), raised with corn cob and a 10% addition of essential oil (CC10), and raised with corn cob and a 20% addition of essential oil (CC20). These samples were compared with a conventional feed substrate, wheat bran, which was used as a control sample. Just as percentages of essential oils were added to the previous substrates, they were also added to the control sample. Therefore, the larvae used as control samples were raised on plain wheat bran (WB0), wheat bran with 10% added essential oil (WB10), and wheat bran with 25% added essential oil (WB25). Regarding the results, the highest protein content was recorded in RB0 (53.4%), WB10 (52.1%), and CC10 (51.3%). The addition of 20% essential oil seemed to have no favorable effect on any of the samples, as their protein content appeared to decrease. In fact, for the TM larvae in the CC25 sample, the protein profile decreased by 15.54%, compared to the highest value recorded in the larvae that consumed corn. The highest ash content was again recorded in the larvae that consumed rice bran. However, it was necessary to add 20% essential oil to ensure a 6.1% metal content. Additionally, the addition of 10% essential oil to the WB and CC samples seems to have no favorable effect on the ash content in the larvae, as the CC10 sample showed a 2.5% content. This value was reduced by 112% and 108% compared to the CC0 and CC25 samples, respectively. Furthermore, the WB10 sample reduced ash content by 14.58% and 8.33% compared to the WB10 and WB20 samples, respectively. It can be concluded, therefore, that rice bran can be a favorable and nutritious alternative substrate for the rearing of TM larvae.

The effect of maize by-products on the growth and nutritional value of TM larvae has been studied again one year later by Naser El Deen et al. [119]. Specifically, four different particle sizes of feeding substrates were studied, that is, 0–0.8 mm, 0.8–2 mm, 2–3 mm, and 3–4 mm, but in terms of growth and nutritional value of TM larvae, only the first (0.8–2 mm) and the last (3–4 mm) were analyzed. Therefore, there were TM larvae reared with 0.8–2 mm wheat bran—sample 1, TM larvae reared with 3–4 mm wheat bran—sample 2, TM larvae reared with grounded corn kernel 0.8–2 mm—sample 3, and TM larvae reared with grounded corn kernel 3–4 mm—sample 4. Following the outcomes of the investigation, in sample 1, the weight of each larva fluctuated around 126 mg, whereas in sample 2, featuring wheat bran with a larger particle size, the weight decreased to 101 mg. Similar findings were found in samples 3 and 4, with sample 4 exhibiting severely underdeveloped larvae weighing 38 mg. Sample 3 demonstrated relatively satisfactory growth results, with the weight of each larva reaching approximately 110 mg. Thus, in the present study, it is observed from the results that corn substrate is not a highly supportive substrate, especially in high particle size, for rearing TM larvae since the weight of larvae from samples 3 and 4 is less than the corresponding samples 1 and 2. Moreover, sample 4 could be deemed unsuitable. Furthermore, it is implied that the smaller the feed particles, the better the larval growth because, presumably, TM larvae can consume their diet more easily in powder form. 

Rumbos et al. [120] examined the suitability of different diets of cereal by-products in terms of their suitability for rearing the TM insect. According to this research, 400 adult TM individuals were placed on different substrates as a feed when the insects were still less than 1 month old (barley flakes—sample 1, corn starch—sample 2, millet flakes—sample 3, millet grains—sample 4, oat flakes—sample 5, rye flakes—sample 6, vittes (wheat by-product)—sample 7, and rapeseed meal—sample 8). All samples were allowed to feed for 10 weeks, and adult mortality, number of larvae, and individual weight of each larva were examined. Adult mortality showed promising results, as the highest mortality was observed in sample 7, which reached 5%. Samples 1, 2, 3, and 6 recorded the same mortality rate of 3.3%. In addition, sample 8 showed a mortality rate of 1.7%, but the best results were recorded in samples 4 and 5, where zero mortality occurred. Regarding the number of alive larvae from adult oviposition within the 10-week period, it was recorded: about 104 larvae in sample 2, about 103 larvae in sample 3, and about 130 larvae in sample 7. In addition, the number of larvae detected in the other samples ranged from 7 (sample 1) to about 70 (sample 5). Thus, although zero mortality for adult insects occurred in sample 5, the number of larvae did not reach the highest values, as was the case in sample 4, where only 53 TM larvae were found alive. The highest larval weight was found in sample 6, where it reached 8.3 mg per larva, while in sample 7, the larvae (which were the highest of all samples) had a weight of 1.9 mg each. Although it cannot be said with certainty which of the samples is most suitable for the rearing of TM larvae, it is certain that all of the substrates can be used as oviposition and feeding substrates for adult TM individuals.

One year later, Rumbos et al. [121] examined the effect of four cereals added to the diet of TM larvae on the growth of these larvae. Concretely, triticale (*Triticum* sp. × *Secale cereale*) by-product—sample 1, barley (*Hordeum vulgare*) by-product—sample 2, durum wheat (*Triticum durum*) by-product—sample 3, and oat (*Avena sativa*) by-product—sample 4 were used as dietary substrates. An additional sample was also created, the control sample, where TM larvae were reared with wheat bran:yeast in a 9:1 ratio. At the end of rearing, each larval from the control sample weighed approximately 134 mg, while each larval from sample 1 weighed 108 mg, each larva from sample 2 weighed approximately 60 mg, each larva from sample 3 weighed approximately 64 mg, and each larva from sample 4 weighed 90 mg. Comparing all the samples with the control sample, it is noted that in each sample, the weight of a single larval is reduced by 24.07, 123.33, 109.38, and 48.89% for samples 1, 2, 3, and 4, respectively. Therefore, it is concluded that the sub-products of these cereals are not beneficial for TM larvae, despite triticale sub-products that could be promising new feeding substrates, especially perhaps with some admixtures with the conventional feed of TM larvae.

Thirteen diets consisting of various proportions of chicken feed, rapeseed meal, wheat bran, and willow-leaf sunflower were used as a feeding substrate for TM larvae and tested for their influence on larval development days (from hatching to pupation) and survival during this period, by Bordiean et al. [122]. The diet samples, in detail, were as follows: 100% chicken feed, (control sample), 100% rapeseed meal (sample 1), 75% rapeseed meal mixed with 25% chicken feed (sample 3), 50% rapeseed meal mixed with 50% chicken feed (sample 4), 25% rapeseed meal mixed with 75% chicken feed (sample 5), 100% wheat bran (sample 6), 75% wheat bran meal mixed with 25% chicken feed (sample 7), 50% wheat bran mixed with 50% chicken feed (sample 8), 25% wheat bran mixed with 75% chicken feed (sample 9), 100% willow-leaf sunflower (sample 10), 75% willow leaf sunflower mixed with 25% chicken feed (sample 11), 50% willow-leaf sunflower mixed with 50% chicken feed (sample 12), and 25% willow leaf sunflower mixed with 75% chicken feed (sample 13). The larvae of all samples exhibited high viability, as they all exceeded 95% except those reared with sample 6, where they recorded viability of about 92%. While the highest viability was recorded in the larvae reared, sample 13 reaching almost 98% (97.7%), 1.77% higher viability compared to the control sample (96%) and 6.20% compared to sample 6 (lower viability). Also, all the larvae completed their growth in about 76 days, except for the larvae reared with samples 10 and 11, which completed their growth in about 115 and 82 days, respectively. It is worth noting that the larvae of sample 9 showed both high viability (95.8%) and the shortest growth time (73 days). Therefore, it could be suggested that the combination of 25% wheat bran mixed with 75% chicken feed is an ideal substrate for the mass production of larvae in a very short period of time.

Rumbos et al. [49] sought to exploit various by-products from Greek agricultural cultivations in 2022, such as barley and oats. They reared larvae TM with four different by-products, namely: barley class I by-product—sample 1, barley class II by-product—sample 2, and oat by-product—sample 3. In all samples, the larvae were allowed to undergo a 12-week rearing period and were subsequently evaluated for their survival and content of nitrogen and lipids. Across all samples, the ones that exhibited substantial viability consistently exceeded 90%, with the lowest viability recorded in TM larvae reared with the control sample and sample 2 (90% in both cases). Furthermore, the highest viability was observed in larvae reared with sample 1 (96.7%). Regarding the nitrogen content of TM larvae, values ranged from 8.4 (TM larvae reared with samples 2 and 3) to 9.1% (TM larvae reared with sample 1). Additionally, the ones reared with the control sample exhibited a nitrogen content of 8.5%. The highest lipid content was recorded in TM larvae from samples 2 and 3, with values of 37.5 and 34.4%, respectively. Conversely, larvae from sample 1 exhibited the lowest lipid content (27.1%), representing a difference of 27.73, 21.22, and 9.06% compared to those from sample 2, sample 3, and the control sample, respectively. Therefore, all the aforementioned cereals could be effectively used for the mass production of TM larvae, yielding superior results compared to the control sample.

### 4.4. Legume By-Products as Feed Substrate for Tenebrio molitor (TM) Larvae

Protein-based dietary substrates have been reported as leading to greater insect growth in TM larvae [123]. This was demonstrated by Rumbos et al. [121], who studied five different legume by-products as feeding and growth substrates for TM larvae and compared them with a control sample. The tested substrates were vetch (*Vicia sativa*) by-product (24.9% crude protein content), pea (*Pisum sativum*) by-product (28.2% crude protein content), lupin (*Lupinus albus*) by-product (33.5% crude protein content), lentil (*Lens culinaris*) by-product (20.8% crude protein content), lucerne (*Medicago sativa*) by-product (13.3% crude protein content), and broad bean (*Vicia faba*) by-product (27.3% crude protein content). The larvae in the control sample had wheat bran:yeast as a food substrate in a 9:1 ratio, which is one of the most prevalent food substrates for TM larvae [63]. TM larvae reared in the control sample had a final weight of 134 mg/larva, while the larvae reared on a lupin diet had a final weight of 130 mg/larva, that is, 3.08% less weight. With respect to the other samples, the weight of each larva was 60, 70, 60, 80, and 63 mg in the larval samples reared with vetch, pea, lupin, lentil, lucerne, and broad bean by-products, respectively. Thus, it is perceived that lupin, which is an untapped source of nutrients [124], can be easily used for rearing TM larvae without significantly affecting their growth.

Two additional leguminous crops were investigated in terms of their suitability for the rearing of TM larvae. The samples used pertained to two classes of peas, namely pea by-product (class I)—sample 1 and pea by-product (class II)—sample 2, as well as two classes of vetch: vetch by-product (class I)—sample 3 and vetch by-product (class II)—sample 4 [49]. For result validity, a control substrate (100% wheat bran) was also examined. The larvae were reared for the same duration across the various substrates (12 weeks) and were evaluated for their suitability through the measurement of larvae viability at the end of the rearing period. The control sample exhibited high viability of TM larvae (90%), while sample 3 demonstrated the highest viability at 96.7%. Unfortunately, the remaining samples were deemed unsuitable for the rearing of TM larvae, as they displayed exceptionally low viability, not exceeding 17.5%. Samples that demonstrated high viability also exhibited significant quantities of nitrogen and lipids. Specifically, larvae from sample 3 displayed 9.4% nitrogen content (an increase of 9.5% compared to larvae from the control sample) and 22.6% lipid content (a decrease of 24.16% compared to larvae reared with the control sample). It is thus inferred that sample 3 could be employed for mass production of larvae, augmenting viability and consequently enhancing TM larvae production.

### 4.5. Crop By-products as Feed Substrate for Tenebrio molitor (TM) Larvae

Rumbos et al. [49] investigated the effect of three different by-products from various crops, namely cotton (fiber crop), sugar beet (tuber crop), and sunflower (oilseed crop), as dietary substrates for TM larvae and compared them with the traditional dietary substrate for TM larvae, namely wheat bran. The samples/by-products used were: cotton cake (sample 1) and cottonseed meal (sample 2) as cotton by-products, sugar beet pulp meal (sample 3) as a sugar beet by-product, and sunflower meal (sample 4) as a sunflower by-product. The samples were left for larvae to feed on for 12 weeks, and their survival rates were assessed during this period. The control group larvae exhibited high viability, hovering around 90%, while the other samples recorded very low survival rates to the extent that they could not be considered suitable dietary substrates. Specifically, larvae reared on samples 1 and 2 exhibited viability rates of 27.5% and 19.2%, respectively. Furthermore, larvae reared on the remaining samples 3 and 4 both recorded the same viability rate of 2.5%.

### 4.6. Fruit Tree By-Products as Feed Substrate for Tenebrio molitor (TM) Larvae

Olive trees and their by-products were the only fruit trees that attracted the interest of researchers for the rearing of TM larvae. Olive oil has a huge commercial value, especially in Mediterranean countries [125,126]. Specifically, according to the Food and Agriculture Organization of the United Nations (FAO), 2.7 million tons of olive oil were produced annually worldwide, with Spain, Italy, and Greece being the leading producing countries with 35.20, 23.10, and 16.10% of the total production, respectively [127,128]. The treatment of olives for oil production generates huge volumes of solid residues, called olive pomace [129]. Several attempts have been carried out to exploit this by-product, both to reduce the ecological impact and to exploit a nutrient product that is a waste product. Olive pomace is a source of bioactive compounds such as polyphenols, dietary fibers, and tocopherols, which help in the production of biological detoxification, and therefore it is often used as animal feed [130,131,132]. Ruschioni et al. [62] studied this by-product in different proportions in their diet in order to evaluate the effect on the growth, viability, and weight of TM larvae. In addition, the effect of olive pomace supplementation on the nutritional value of larvae after rearing was also studied. In total, the five feeding substrates tested were as follows: 100% organic wheat flour (sample 1), 100% organic wheat middlings (sample 2), 75% wheat middlings mixed with 25% organic olive pomace (sample 3), 50% wheat middlings mixed with 50% organic olive pomace (sample 4), and 25% wheat middlings mixed with 75% organic olive pomace (sample 5). TM larvae completed their rearing (conversion to pupae) in 137, 109, 112, 112, 140, and 147 days for samples 1, 2, 3, 4, and 5, respectively. The viability of these larvae in samples 1, 2, 3, 4, and 5 reached 79, 85, 78, 54, and 58%, respectively, while the weight of the respective samples was recorded at 0.11, 0.13, 0.13, 0.08, and 0.07 g. Regarding their nutritional profile, the protein content does not seem to be promoted by the presence of olive pomace since, as its content increases, the crude protein content of the larvae decreases. In particular, larvae reared with samples 2, 3, 4, and 5 showed 50.14, 47.58, 39.39, and 38.05%, respectively, that is, protein among larvae reared by feeding olive pomace decreased by 5.38, 27.29, and 31.77% compared to those reared with sample 2. However, comparing the larvae of samples 3, 4, and 5 with the larvae reared with sample 1, the most prevalent insect feed, there was an increase in protein content in them by 20.59, 4.09, and 0.71%, respectively. Therefore, it may be said that the addition of olive pomace to the insects’ diet neither favors their growth and development nor enhances their protein profile.

Andreadis et al. [47] also focused on the olive tree and studied the residues of the olive tree crop as a possible feeding substrate for TM larvae. The addition of essential oil was done at 10 and 20%, so the larvae were reared with 100% olive oil plant residue substrate (sample 1), 10% essential oil added to olive oil plant residues (sample 2), and 20% essential oil added to olive oil plant residues (sample 3). In addition, larvae reared on a conventional rearing diet of wheat bran, which was used either as such (sample 4) or with the addition of both 10 (sample 5) and 20% essential oil (sample 6), were also studied. The crude protein content of samples 4, 5, and 6 was 51.2, 52.1, and 50.6%, respectively, while in samples 1, 2, and 3, the content was 57.5, 56.0, and 42.8%, respectively. Thus, it appears that olive by-products can greatly favor crude protein content, especially when used as such, since they showed a 12.30% enhanced protein profile compared to the corresponding control sample (WB0). Furthermore, the ash content of larvae reared on rice bran was also found to be highly increased. Sample 6 showed 13.1% ash, sample 5 showed 9.8% ash, and sample 4 showed 6.7% ash. This may be due to the fact that the substrates were very rich in minerals/ash, so the larvae were able to absorb through consumption of their food what this diet contained. It can be seen, therefore, that the residues of the olive tree crop can be a highly nutritious and advantageous feeding substrate for TM larvae.

### 4.7. Εdible Fungi by-Products as Feed Substrate for Tenebrio molitor (TM) Larvae

The term “fungi” refers to the fleshy, spore-bearing fruiting body of a fungus, typically produced above ground on soil or its food source [133]. Mushrooms have been consumed since 4000 B.C. in Mycenaean Greece [134], and today, many countries are capable of producing and distributing large quantities of edible fungi worldwide [135]. Like all other by-products of widely consumed food items, mushroom by-products remain underutilized, representing waste. Li et al. [134] sought to identify novel means of utilizing the by-products of edible mushrooms. For this reason, they investigated by-products from five species of mushrooms (*Auricularia cornea*, *Lentinus edodes*, *Pleurotus eryngii*, *Pleurotus citrinopileatus*, and *Pleurotus ostreatus*) as dietary substrates for TM larvae (the end of the experiment was marked by the appearance of pupae in the dietary substrate). In a preliminary experiment, all by-products were tested as the sole feed source for the larvae, with the addition of a moisture source (cucumber slices). Additionally, two control groups were used for experiment evaluation: TM larvae reared on 100% wheat bran and 100% rice bran. The results were not very promising, as the only dietary substrates on which the larvae were able to survive were the two control substrates and the substrate containing *L. edodes*. Nevertheless, the TM larvae that survived by consuming mushroom by-products exhibited very low survival rates of 36.7% and a very low larval weight of 0.53 mg per larva. It should be noted at this point that the larvae reared on wheat bran exhibited a 96% survival rate and a weight of 6.84 mg per larva, while those reared on rice bran showed a 96% survival rate and a weight of 3.56 mg per larva. For this reason, further investigations were conducted with various proportions of *L. edodes* in the diets, as well as with the two control groups separately. Consequently, five diets consisting of wheat bran mixed with 0, 30, 40, 50, 60, and 70% of *L. edodes* by-products, and rice bran mixed with 0, 20, 30, 40, 50, and 60% *L. edodes* by-products were created, along with an additional moisture source (watermelon rinds). As expected, as the proportion of by-products in the dietary substrates increased, the viability and weight of the TM larvae decreased. The most tolerable percentages of *L. edodes* by-product addition to rice bran were up to 30%, while for wheat bran, it was up to 60%. Therefore, edible fungi are not particularly favorable for rearing TM larvae, especially at high proportions.

### 4.8. Beverage By-Products as Feed Substrate for Tenebrio molitor (TM) Larvae

Coffee is the most widespread beverage worldwide and ranks as the second-largest commercial product [136]. Among the various by-products produced and discarded annually are the residues generated after roasting and processing coffee beans, that is, spent coffee grounds (SCG), which reach approximately 6 million tons annually [137,138]. Intending to reduce waste and promote a circular economy aligned with broader sustainability goals, Kotsou et al. used SCG as a nutritional substrate for TM larvae [139]. Specifically, two percentages of SCG (10 and 25% *w*/*w*) were mixed with wheat bran (where plain wheat bran was used as a control substrate) and studied both the viability and growth as well as the nutritional composition, including proteins, lipids, carbohydrates, ash, carotenoids, vitamins A and C, and polyphenols. A positive outcome is that the viability and growth of TM larvae fed with SCG were similar, almost better than those of the control TM larvae. Additionally, a significant enhancement was observed in the protein content of TM larvae fed with SCG compared to the control sample, recording values of 32.59%—control sample larvae, 47.34%—SCG 10 sample TM larvae, and 42.65%—SCG 25 sample TM larvae. Another notable result is that as the amount of SCG in the substrate increased, so did the nutritional value of the TM larvae. For example, the content of vitamin C showed an increase of 81.28% and vitamin A of 822.79%, while in polyphenols, the profile of the larvae showed an increase of 29.01% compared to the control. Furthermore, the oil extracted from them was also studied, presenting evaluated nutritional value and high resistance to oxidation. The utilization of SCG as dietary supplements for TM larvae shows great promise, offering a sustainable method to improve their nutritional quality.

### 4.9. Mixed By-Products as Feed Substrate for Tenebrio molitor (TM) Larvae

The use of potato substitutes as an alternative diet for TM larvae has attracted so much interest from scientists that, besides being studied as such, it has also been studied in various mixtures with other substitutes. In particular, Oonincx et al. [140] examined three diets: high protein combined with high fat content (60% spent grains, 20% beer yeast, and 20% cookie remains—sample 1), high protein combined with low fat content (50% beer yeast, 30% potato steam peelings, and 20% beet molasses—sample 2), and low protein combined with low fat (30% potato steam peelings, 20% beet molasses, and 50% bread—sample 3) for their effect on the growth, development, and nutritional value of TM larvae. The larvae of samples 1, 2, and 3 besides being compared with each other, were compared with two control samples (control 1 and control 2) reared on an unknown feeding substrate but of almost the same nutritional value. Regarding the growth and development of TM larvae, it appears that the high protein and fat diet (sample 1) had the highest growth and development among all the samples. In particular, the TM larvae of sample 1 had 79% survival rate and completed their development (conversion to pupae) in 116 days, the TM larvae of sample 2 had 67% survival rate and completed their development in 144 days, the TM larvae of sample 3 had 52% survival rate and completed their development in 227 days. Additionally, the larvae of the two control samples, control 1 and control 2, had 84% and 34% survival rate and completed their development (conversion to pupae) in 145 and 151 days, respectively. Hence, a high-protein and high-fat substrate diet is highly conducive to larval growth. Interesting results of larvae content in crude protein, phosphorus, and total fatty acids are found by examining the results of larvae reared on various dietary substrates. On a general level, the larvae of sample 1 showed the highest percentage of protein and phosphorus compared to the other samples tested, while the larvae of control 2 showed the highest percentage of fatty acids. In detail, the protein profile (PP), phosphorus (PA), and total fatty acid (FAA) content of the larvae hatched with the different samples yielded the following values. The larvae of sample 1 showed values of 53.6% PP, 8.9 g/kg PA, and 26.5% FAA, and the larvae of sample 2 showed values of 53.5% PP, 8.8 g/kg PA, and 23.0% FAA, that is, the only significant difference was in fatty acids, where sample 1 had 15.2% increased fatty acid content. On further comparison, the TM larvae of sample 1 compared with the more nutritious control sample (control 1) showed 2.29% higher PP, 8.89% lower PA, and 1.89% lower FAA. So, it is concluded that, depending on the characteristics needed to impart to TM larvae, the appropriate diet will be given. In the case of the PP increase, diet 1 was the most suitable, giving at the same time more advantageous growth and development results.

Stull et al. [24] decided to compare two mixtures of by-products, in order to evaluate their effect on the development of TM larvae in terms of their final weight, protein profile, and iron content. These diets consisted of wheat bran, oats, brewer’s yeast (50:45:5% by weight)—control sample and organic corn meal, organic soy flour, dry stover (chopped) (30:30:40% by weight)—sample for evaluation. In the control sample, the larvae showed a weight of 128.94 mg/g larva, while in the sample to be evaluated, the larvae showed a weight of 89.35 mg/g larva, that is, in the control sample, the weight of the larvae was increased by 44.31%. However, although the difference in larval weight was significant, this difference was not reflected in the larval protein content. The larvae reared from the control sample had a crude protein content of 19.33%, while the larvae consuming the test bait recorded a content of 19.93%. Similar results were recorded in the iron content of the larvae, where the control TM larvae had 0.0157 mg iron/g larval while the TM larvae of the sample to be evaluated had 0.0153 mg iron/g larva, a difference of 2.61% with the control sample larvae predominating in content.

The effect of a feed mixture on the survival, growth, and protein value of TM larvae was investigated by Riudavets et al. [141]. The dietary substrate of TM larvae consisted of brewer’s spent grain (dried) mixed with brewer’s spent yeast (dried) and apricot in a ratio of 3.5:1:5. In order to have a control sample, TM larvae were also reared on a standard diet containing whole flour wheat, wheat bran, and pet food (ultima dog food with chicken, Affinity Petcare) in a ratio of 3.3:2.5:1. As far as viability is concerned, TM larvae reared with the control sample (sample 1) reached 92%, while TM larvae reared with the test sample (sample 2) showed a very low viability of 24.8%. Also, the weight of sample 2 was 99 mg, a 58.08% lower weight compared to sample 1 (156.5 mg). Although the viability and growth of sample 2 were not at promising levels, a high crude protein content of 43.60% was recorded in this sample, which was 10.55% lower than the protein content of sample 2. So, a mixture of brewer’s spent grain (dried) mixed with brewer’s spent yeast (dried) and apricot in the ratio of 3.5:1:5 cannot be a promising alternative diet for the larvae due to their greatly reduced viability.

Two diets for TM rearing, consisting of vegetable and cereal by-products, were evaluated by Montalbán et al. [70] in order to examine their effect on the growth, development, and nutritional value of these larvae. The first sample (sample 1) was the larvae reared on a diet consisting of 30% courgette (*Cucurbita pepo*) remains, 5% tigernut (*Cyperus scelentus*) pulp, 10% brewer’s spent grains, 40% bread remains, 10% brewer’s yeast, and 5% rice straw, containing 20% starch content and 21% protein content. Also, a second sample (sample 2) was generated, which consisted of TM larvae reared with 25% courgette remains, 5% tiger nut pulp, 25% brewer’s spent grains, 20% bread remains, 20% brewer’s yeast, and 5% rice straw and contained 20% starch content and 26.3% protein content. The experimental procedure was terminated, and all experimental procedures were performed when at least one larva was converted to pupae in each feeding substrate. Therefore, growth and development for samples 1 and 2 were completed in about 121 and 91 days, respectively, while the larval mortality recorded was 13.63 and 14.54%, respectively. In addition, the weight of larvae was also measured, which was 112.99 mg in total (0.65 mg per larva) for sample 1 and 168.69 mg in total (0.69 mg per larva) for sample 2. With the results obtained so far, both feeding substrates appear to be appropriate for mass production of TM larvae, with sample 2 being superior in terms of rapid growth and development. Subsequently, the samples were evaluated for their protein content, with sample 1 having 49.07% crude protein content and sample 2 recording 52.46%. Therefore, in both samples, the protein content is very elevated, with sample 2 showing an increase in 6.46% protein content. The samples were also analyzed for macrominerals. Ca, P, Na, Mg, and K were identified, quantified, and detected at 0.09, 0.83, 0.22, 0.21, and 1.22% for sample 1 and 0.11, 0.86, 0.19, 0.24, and 1.12% for sample 2, respectively. In addition, the phyla of the microbiota sequence of each sample were also identified and quantified. The total of seven phyla, such as Tenericutes, Proteobacteria, Firmicutes, Cyanobacteria, Fusobacteria, Bacteroidetes, and Actinobacteria, were recorded in percentages of 70.08, 17.99, 7.89, 1.23, 2.09, 0.51, and 0.01% for sample 1 and 13.41, 42.84, 28.71, 8.46, 5.23, 0.71, and 0.30% for sample 2, respectively. It is therefore concluded that a large number of microbes develop in sample 2. Therefore, the feeding substrate of sample 1 can be considered a suitable and enhanced feeding substrate since it favors growth and development while enriching the nutrients of TM larvae. Nevertheless, a high growth rate of Tenericutes, which are pathogenic and/or mutualistic symbioses in the gut of their host species, is discerned [142].

Various organic waste products derived from vegetables were examined as an alternative and economically viable feed substrate for TM larvae [143]. The vegetable by-products used consisted of composite peels from 10% onions, 25% potatoes, 25% sweet potatoes, 30% carrots, and 10% cucumbers, with a total water content of 91.4%. These were blended with conventional chicken feed in a 9:1 ratio. A control sample of 100% conventional chicken feed (rich in protein) was employed for comparison. Larvae reared with the control sample exhibited a more nutrient-dense profile (as anticipated due to the protein-rich composition of the diet), with crude protein content reaching 47.18 g/kg, representing a 1.87% increase compared to larvae reared with vegetable by-products (46.30 g/kg). Additionally, larvae reared with the control sample displayed a higher mineral content (3.08 g/kg), an increase of 2.27% compared to larvae reared with the examined sample (3.01 g/kg). Finally, larvae reared with vegetable by-products exhibited a fiber content of 8.01 g/kg, whereas those from the control sample registered only 7.44 g/kg, indicating a 7.11% lower content. Although larvae reared with the examined substrate did not demonstrate enhanced nutritional value compared to those reared with the control sample, they exhibited a significantly higher content of crude protein, fiber, and ash. Therefore, the examined diet could effectively substitute conventional feeds for TM larvae, resulting in the production of nutrient-rich TM larvae through the consumption of a zero-cost diet.

Bordiean et al. [144] have also studied various nutritional substrates for rearing TM larvae and their effect on the nutritional value of TM larvae. In particular, they investigated the effect of mixing several cereal sub-products with the conventional TM larval food, bran, creating five different dietary substrates. For this, 100% wheat bran was used as a control, followed by sample 1—70% wheat bran mixed with 30% rye bran, sample 2—70% wheat bran mixed with 30% rapeseed meal, sample 3—70% wheat bran mixed with 30% rapeseed cake, sample 4—70% wheat bran mixed with 30% flax cake, and sample 5—70% wheat bran mixed with 30% *Silybum marianum* cake. Larvae reared with sample 4 showed the highest protein percentage (53.4%), noting an increased protein profile by 11.48, 8.32, 1.71, 4.91, and 2.69% compared to control, 1, 2, 3, and 5, respectively. Also noteworthy is that all the samples showed a higher protein percentage than the control sample, demonstrating that all the sub-products can favor the nutritional profile of the larvae even at 30% in combination with wheat bran. In addition, sample 4 greatly enhanced the ash and fiber content of TM larvae, recording values of 5.19% and 7.31%, respectively. This result is not unexpected since flaxseed cake is a rich source of proteins and fiber [145] and appears highly promising for the rearing of TM larvae. An overview of all the examined diets of TM as well as the protein content of TM can be seen in Table 1.

## 5. Conclusions and Future Perspectives

The review emphasizes the potential use of agri-food waste as a food source for mealworms (*Tenebrio molitor* larvae) to promote sustainable practices in the agri-food industry. This approach leverages the potentially rich nutritional content present in these waste materials, including proteins, carbohydrates, lipids, vitamins, and bioactive compounds vital for insect development. It not only supports environmentally responsible waste management but also enhances the economic sustainability of TM larvae rearing, achieving one of the 17 SDGs concerning “sustainable consumption and production patterns”. The inclusion of agricultural food waste in the diet of mealworms either demonstrates significant benefits, leading to increased mass production, cost reduction, and overall improved nutritional quality (such as tomato pomace, rice bran, and orange albedo), or indicates that they cannot be compared and substituted for conventional feeds for TM larvae (cotton by-products and mushrooms). The significant potential of repurposing agri-food waste for mealworm rearing, with far-reaching implications for sustainable protein production for human consumption, as well as its utility as a valuable feed ingredient in the livestock and aquaculture sectors, highlights a more ecologically sound and economically viable agri-food landscape. The integration of such innovative practices holds promise for a shift towards a more ecologically sound and economically viable agri-food landscape. Furthermore, several key directions for future research or application were emphasized. First, the optimization of waste selection. Research has focused on understanding specific agricultural food waste materials that are most suitable for mealworm rearing in order to ensure maximum production of mealworm larvae. While the use of these substrates may not be applicable on an industrial scale, only in laboratory settings, this research will establish a solid foundation for the implementation of TM larval rearing using the most suitable food waste. Second, there are strategies for creating enhanced nutrition. Future studies could explore targeted approaches to improving the nutritional composition of mealworms through their diet. This may include supplementing specific nutrients to maximize the availability of essential compounds, resulting in the production of mealworms with even higher nutritional value, without the use of additional chemicals. Finally, the integration of circular economy principles. Exploring broader applications of agri-food waste within a circular economy framework is a mature avenue for exploration. Research efforts could extend towards valorizing waste streams for additional uses, thereby maximizing resource efficiency and minimizing environmental impact.

In conclusion, the future of utilizing agri-food waste for mealworm rearing is promising, with various research directions offering potential advancements in sustainability, nutrition, and waste management. Addressing these future perspectives may unlock the full potential of this innovative approach, contributing to a more resilient and environmentally conscious industry that exploits nutritional food wastes for the mass production of mealworm larvae as an alternative, economical, and environmentally friendly source of protein and additional nutrients for human consumption.

## Figures and Tables

**Table 1:** Protein content (%) of *Tenebrio molitor* larvae reared with several by-product diets.

By-Products	Diets	Protein Content (%)	Ref.
Fruits	Wheat bran 100% (control)	~50.00	[84]
	Wheat bran 73% and tomato pomace 27%	47.30	
	Wheat bran 59% and tomato pomace 41%	49.20	
	Tomato pomace 100%	42.00	
	Bread 100% (control)	41.51	[91]
	Watermelon rinds 100%	43.38	
	Banana peels 100%	38.53	
	Wheat bran 100% (control)	32.25	[89]
	Wheat bran 90% and orange albedo 10%	36.50	
	Wheat bran 82.5% and orange albedo 17.5%	40.18	
	Wheat bran 75% and orange albedo 25%	44.20	
Vegetables	Wheat bran 100%	51.20	[47]
	Wheat bran mixed with 10% essential oil of residues from medical plant (RMP)	52.10	
	Wheat bran mixed with 20% RMP	50.60	
	Potato peels 100%	59.50	
	Potato peels mixed with 10% RMP	44.10	
	Potato peels mixed with 20% RMP	43.60	
Grains	Brewer’s spent grains 100%	17.36	[110]
	Brewer’s spent grains 50% and 50% biscuits	17.65	
	Wheat bran 50% oats 45% and brewer’s yeast 5% (control)	19.33	[24]
	Organic maize stover dry (chopped) (100%)	15.17	
	Wheat bran 100% (control)	29.31	[115]
	Brewer’s spent grain 100%	18.56	
	Distillers dried grain 100%	29.31	
	Wheat bran 50% and brewer’s spent grain 50%	14.43	
	Wheat bran 50% and distillers dried grain 50%	22.30	
	Wheat bran 100% (control)	19.57	[117]
	Brewer’s spent grain	22.45	
	Wheat bran 100% (control)	51.20	[47]
	Wheat bran mixed with 10% RMP	52.10	
	Wheat bran mixed with 20% RMP	50.60	
	Rice bran 100%	53.40	
	Rice bran mixed with 10% RMP	47.70	
	Rice bran mixed with 20% RMP	47.50	
	Corn cob 100%	50.70	
	Corn cob mixed with 10% RMP	51.30	
	Corn cob mixed with 20% RMP	44.40	
Fruit tree	Organic wheat flour 100%	37.78	[62]
	Organic wheat middlings 100%	50.14	
	Wheat middlings 75% and organic olive pomace 25%	47.58	
	Wheat middlings 50% and organic olive pomace 50%	39.39	
	Wheat middlings 25% and organic olive pomace 75%	38.05	
	Wheat bran 100% (control)	51.20	[47]
	Wheat bran mixed with 10% RMP	52.10	
	Wheat bran mixed with 20% RMP	50.60	
	Olive oil plant residues 100%	57.50	
	Olive oil plant residues mixed with 10% RMP	56.00	
	Olive oil plant residues mixed with 20% RMP	42.80	
Beverages	Wheat bran 100% (control)	32.59	[139]
	Wheat bran mixed with 10% Spent coffee grounds	47.34	
	Wheat bran mixed with 10% Spent coffee grounds	42.65	
Mixed	Spent grains 60% beer yeast 20% and cookie 20%	53.60	[140]
	Beer yeast 50% potato steam peelings 30% and beet molasse 20%	53.50	
	Potato steam peelings 30%, beet molasses 20%, and bread 50%	47.50	
	Wheat bran 50%, oats 45%, and brewer’s yeast 5% (control)	19.33	[24]
	Organic corn meal 30%, organic soy flour 30%, and dry stover (chopped) 40%	19.93	
	Whole flour wheat, wheat bran, and pet food (3.3:2.5:1) (control)	48.20	[141]
	Brewer’s spent grain (dried) mixed with brewer’s spent yeast (dried) and apricot (3.5:1:5)	43.60	
	Courgette (*Cucurbita pepo*) remains 30%, tigernut (*Cyperus scelentus*) pulp 5%, brewer’s spent grains 10%, bread remains 40%, brewer’s yeast 10%, and rice straw 5%	49.07	[70]
	Courgette remains 25%, tigernut pulp 5%, brewer’s spent grains 25%, bread remains 20%, brewer’s yeast 20%, and rice straw 5%	52.46	
	Conventional chicken feed 100% (control)	47.18 g/kg	[143]
	Onions 10%, potatoes 25%, sweet potatoes 25%, carrots 30%, and cucumbers 10% mixed with conventional chicken feed (9:1)	46.30 g/kg	
	Wheat bran 100% (control)	47.90	[144]
	Wheat bran 70% and rye bran 30%	49.30	
	Wheat bran 70% and rapeseed meal 30%	52.50	
	Wheat bran 70% and rapeseed cake 30%	50.90	
	Wheat bran 70% and flax cake 30%	53.40	
	Wheat bran 70% and *Silybum marianum* cake 30%	52.20	

## Data Availability

The original contributions presented in the study are included in the article, further inquiries can be directed to the corresponding author.
